# Monomicrobial non-neutrocytic bacterascites caused by aeromonas hydrophila in a patient with liver cirrhosis

**DOI:** 10.1051/bmdcn/2019090213

**Published:** 2019-05-24

**Authors:** Chen-Sheng Lin, Cheng-Wen Lin

**Affiliations:** 1 Division of Gastroenterology, Kuang Tien General Hospital Taichung 433 Taiwan; 2 Department of Medical Laboratory Science and Biotechnology, China Medical University Taichung 404 Taiwan; 3 Department of Biotechnology, Asia University Wufeng Taichung 413 Taiwan

**Keywords:** Aeromonas hydrophila, Monomicrobial non-neutrocytic bacterascites, Liver cirrhosis

## Abstract

Aeromonas peritonitis is a rare, but serious infection, as associated with spontaneous bacterial peritonitis, peritonitis in chronic ambulatory peritoneal dialysis, and intestinal perforation. Here, we reported a case of monomicrobial non-neutrocytic bacterascites caused by Aeromonas hydrophila (A. hydrophila). The patient, a 57-year-old man who had a history of alcoholic liver disease and chronic hepatitis C-related Child- Pugh class C liver cirrhosis, was admitted to our hospital with fever, dyspnea and a localized wound pain over left ankle. Ascitic fluid analysis demonstrated that ascitic polymorphonuclear cell count was 30 cells/ mm^3^. Empirical antimicrobial treatment with a combination of ceftriaxone and clindamycin were administered. However, the patient died due to fatal septic shock on Day 3. His blood and ascites cultures were positive for A. hydrophila. The case report presents the diagnosis, management, and literature review of Aeromonas monomicrobial non-neutrocytic bacterascites.

## 1. Introduction

Spontaneous bacterial peritonitis (SBP), including monomicrobial non-neutrocytic bacterascites (MNB), is a common and serious complication in patients with liver cirrhosis.[1, 2] MNB usually represents the bacterial infection of ascitic fluid in the absence of inflammatory reaction that the polymorphonuclear cell count is less than 250 cells/mm.[3] *Escherichia coli* and *Klebsiella pneumoniae* are the most common organisms that cause MNB, as responsible for 71% of ascitic fluid isolates in Taiwan.[3] Aeromonas peritonitis is uncommon inSBP, but usually causes a poor prognosis in patients with advanced liver cirrhosis.[4, 5] Here, we presented a case of septic shock due to MNB with the positive blood and ascites cultures for Aeromonas hydrophila (A. hydrophila).

## 2. Case report

A 57-year-old man who had alcoholic liver disease and chronic hepatitis C-related Child-Pugh class C liver cirrhosis was brought to our emergency department by ambulance, exhibiting fever, short of breath and a localized wound pain over left ankle. The patient presented with dyspnea, lower extremity edema and some wounds over bilateral ankles area for 1 day before admission, but had no other symptoms, including a headache, sore throat, cough, and chest, abdominal, and back pain. He had alcohol and chronic hepatitis C- related liver cirrhosis, and took diuretics in the past 5 years. He drank approximately 60 g of alcohol per day for more than 20 years, but recently had been taking a bit more than usual.

**Fig. 1:**
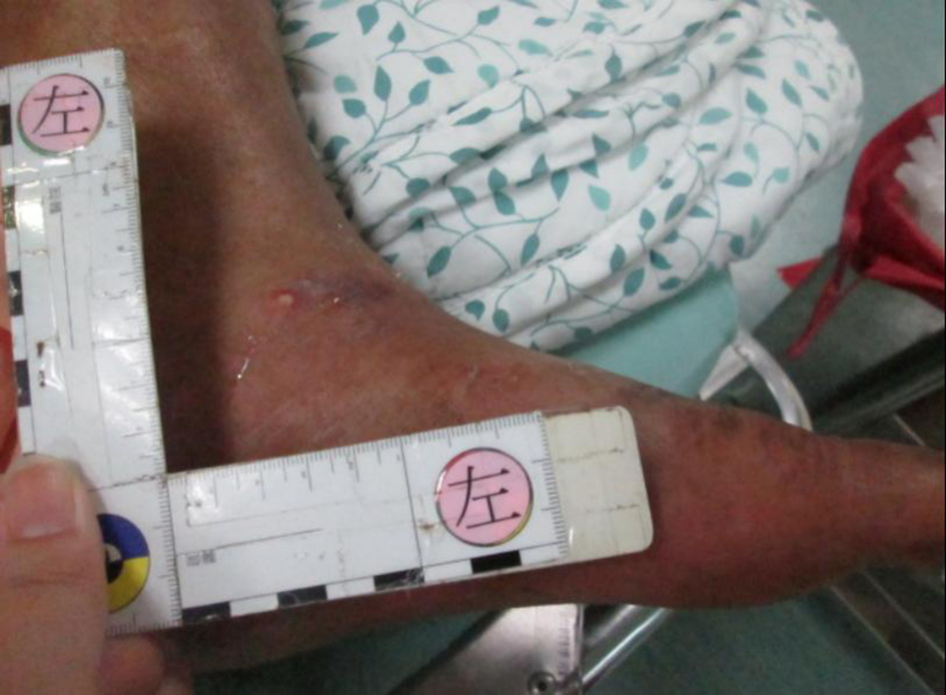
One wound with redness, measures 0.5 × 0.5 cm in left ankle.

**Fig. 2:**
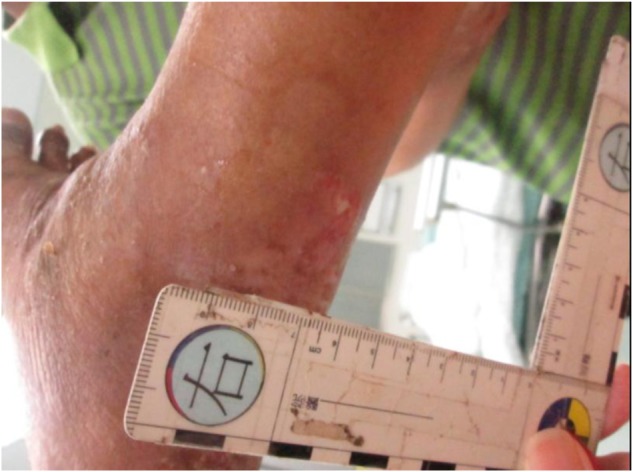
One wound with redness and some echymosis, measures 1.0 × 1.0 cm in right ankle.

**Fig. 3:**
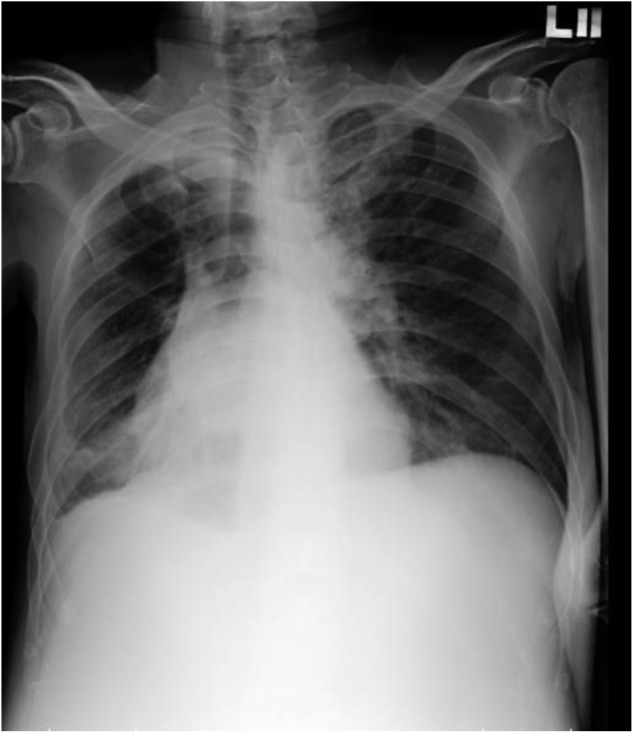
Chest X-ray revealed a lower lobe infiltrate and mild blunting of C-P angle in right side.

His laboratory data 2 weeks before admission were 6.5 mg/ *dl* of total bilirubin, 2.1 g/*dl* of albumin, and 1.73 of international normalized ratio. In addition, he had moderate ascites, which was medically controlled. These findings were indicative of Child- Pugh class C liver cirrhosis. On arrival, he appeared to be in disturbance and distress that was classified as 13 (E3V4M6) on the Glasgow Coma Scale. His vital signs were 132/106 mmHg of blood pressure, 125 beats/minute of pulse rate, 24 breaths/minute of respiratory rate, and 38.4 °C of body temperature. Auscultation of the lung and heart revealed coarse breath sounds and rapid irregular heartbeats. His abdomen was soft and swollen. Two wounds with redness and swelling (measures 0.5 × 0.5 cm, left ankle; measures 1 × 1 cm, right ankle) were found on his lower extremities (Fig. 1 and 2). Chest X-ray indicated a lower lobe infiltration and a mild blunting of C-P angle in the right side (Fig. 3). ECG showed atrial fibrillation.

Laboratory findings also included 8,990/*ml* of white blood cell count with 94% neutrophils and 3% lymphocytes, 11.1 g/*dl* of hemoglobin level, 104,000/*ml* of platelet count, 23 mg/*dl* of blood urea nitrogen, 1.42 mg/*dl* of creatinine, 121 mmol/L of sodium, and 4.6 mmol/L of potassium. Ascitic fluid analysis demonstrated 152 cells/mm^3^ of ascitic white blood cell count and 30 cells/mm^3^ of polymorphonuclear cell count 10 hours later after his arrival of emergency department. Arterial blood gas analysis demonstrated a severe metabolic acidosis (pH: 7.38, pCO2: 20.4 mmHg, pO2: 91.8 mmHg; base deficit: 11.2 mmol/L), which was indicative of septic shock.

Oxacillin treatment under the impression of cellulitis was administered 2 hours after his arrival; two sets of blood cultures were obtained. Rapid fluid resuscitation was performed and immediately developed by tracheal intubation and mechanical ventilation in the intensive care unit. Although there was no significant change of ankle wounds, antibiotic regimen with a combination of ceftriaxone and clindamycin was adjusted due to persistent hypotension unresponsive to fluid resuscitation and high dose vasopressor use and clinical suspicion of *Vibrio vulnificus* related necrotizing fasciitis.

One episode of upper gastrointestinal bleeding with manifestations of fresh blood in the nasogastric tube occurred on Day 2. Blood transfusion with 2 units of packed red blood cells was given. Despite intensive support efforts, the patient died on Day 3 after the sudden change. And after that, his blood and ascites cultures were positive for *A. hydrophila*.

## 3. Discussion

Aeromonas spp., gram-negative, facultative anaerobic bacteria, have been implicated in a variety of human diseases, including acute gastroenteritis, septicemia, skin and soft-tissue infections, hepatobiliary infection, pleuropulmonary infections, meningitis, and hemolytic uremic syndrome.[5, 6] As the report found in southern Taiwan, Aeromonas bacteremia was primary bacteremia (17.6%) and secondary infection comprising peritonitis (29.7%), biliary tract biliary (19.8%) and soft-tissue (13.2%) infections, pneumonia (9.9%), catheter-related bloodstream infection (5.5%), and genitourinary tract infection (4.4%).[5]

Aeromonas species are the third most common pathogens of bacteremia due to gram-negative bacilli in cirrhotic patients in Taiwan.[7] The high prevalence of Aeromonas bacteremia could relate to that the patients with chronic liver diseases and cirrhosis are susceptible to invasive Aeromonas infections if they eat of raw seafood or freshwater ﬁsh, and have the cutaneous exposure to fresh and brackish water or trauma.[4, 7, 8] Wu *et al*. reported that A. sobria (55%) and A. hydrophila (45%) were the causative species of Aeromonas spontaneous bacterial peritonitis.[4] Choi *et al*. compared the clinical manifestations of SBP, indicating that the incidence of Aeromonas infection signiﬁcant increased in warm weather months, as also associated with diarrheal episodes in the group.[8] However, there were no statistically signiﬁcant differences between groups with initial antibiotic therapy and mortality.

Aeromonas and Vibrio are both important water-borne pathogens causing skin and soft-tissue infections (e.g. cellulitis necrotizing fasciitis) in cirrhotic patients in Taiwan. Syue *et al*. reported the differences in clinical manifestations of Aeromonas and Vibrio bacteremia.[9] Vibrio bacteremia was meaningfully correlated with the ingestion of undercooked seafood or the cutaneous exposure to brackish water. Skin and soft-tissue infections were notably more frequent in the group with Vibrio bacteremia than the group with Aeromonas bacteremia. Vibrio and Aeromonas bacteremia were similar to sepsis-related mortality, but Vibrio bacteremia appeared a fulminant course and a shorter period from bacteremia onset to death.

Intravenous third-generation cephalosporins and/or fluoroquinolones are commonly used for treating community-acquired bacteremia without the risk of specific pathogens to develop the antibiotic resistance. Third generation cephalosporins like cefotaxime were known as the effective agents for the SBP treatment. Ciprofloxacin also displayed the effective antimicrobial activity to *in vivo* infection with A. hydrophila.[10] In addition, fluoroquinolone or a combination of cephalosporin and tetracycline analogs was believable alternatives for antimicrobial therapy to systemic Aeromonas infections.

There were some limitations in this case report. First, we did not perform wound cultures neither gram-stained smears from infected wound sites before or after the administration of antimicrobial therapy. Secondly, diagnostic paracentesis was not performed within 3 hours after the administration of antimicrobial therapy. His blood cultures revealed gram-negative bacillus when he was discharged from our hospital later the same day. A repeat paracentesis had no chance to be performed. Thirdly, clinical history of diarrhea symptom and exposure history were not available due to the unconscious status of the patient.

In summary, we stated an unusual case of monomicrobial non-neutrocytic bacterascites caused by Aeromonas hydrophila. Aeromonas peritonitis, although rare, should be considered in cirrhotic patients presenting with diarrheal symptom and profound shock in the warm season. Particularly, paracentesis was recommended for cirrhotic patients with ascites and patients who develop other signs as suggestive of peritoneal infection on hospital admission. Antimicrobial therapy (e.g. third generation cephalosporins) might be given before or immediately after the sampling of blood and ascitic fluid.

## Conflicts of interest statement

The authors wish to disclose no conflicts of interest.
